# Novel transaminases from thermophiles: from discovery to application

**DOI:** 10.1111/1751-7915.13940

**Published:** 2021-10-29

**Authors:** Max Cárdenas‐Fernández, Oliver Sinclair, John M. Ward

**Affiliations:** ^1^ Department of Biochemical Engineering University College London Gower St WC1E 6BT London UK; ^2^ School of Biosciences University of Kent CT2 7NJ Kent UK

## Abstract

Transaminases (TAs) are promising biocatalysts for chiral amine synthesis; however, only few thermophilic TAs have been described to date. In this work, a genome mining approach was taken to seek novel TAs from nine thermophilic microorganisms. TA sequences were identified from their respective genome sequences and their P*fam* were predicted confirming that TAs class I–II are the most abundant (50%), followed by class III (26%), V (16%), IV (8%) and VI (1%). The percentage of open reading frames (ORFs) that are TAs ranges from 0.689% in *Thermococcus litoralis* to 0.424% in *Sulfolobus solfataricus*. A total of 94 putative TAs were successfully cloned and expressed into *E. coli*, showing mostly good expression levels when using a chemical chaperone media containing d‐sorbitol. Kinetic and end‐point colorimetric assays with different amino donors–acceptors confirmed TAs activity allowing for initial exploration of the substrate scope. Stereoselective and non‐stereoselective serine‐TAs were selected for the synthesis of hydroxypyruvate (HPA). Low HPA reaction yields were observed with four non‐stereoselective serine‐TAs, whilst two stereoselective serine‐TAs showed significantly higher yields. Coupling serine‐TA reactions to a transketolase to yield l‐erythrulose (Ery) substantially increased serine conversion into HPA. Combining both stereoselective serine‐TAs and transketolase using the inexpensive racemic D/L‐serine led to high Ery yield (82%). Thermal characterization of stereoselective serine‐TAs confirmed they have excellent thermostability up to 60°C and high optimum temperatures.

## Graphical abstract

Transaminases (TAs) are promising biocatalysts for chiral amine synthesis, however only few thermophilic TAs have been described to date. We have cloned and characterised 94 TAs from nine thermophilic bacteria. Stereoselective and non‐stereoselective serine‐TAs were selected for the synthesis of hydroxypyruvate (HPA) which is a vital donor substrate for transkeolase C‐C bond formation. Low HPA yields were observed with 4 non‐stereoselective serine‐TAs, whilst two stereoselective serine‐TAs showed significant higher yields. Coupling serine‐TA reactions to a transketolase to yield l‐erythrulose (Ery) substantially increased serine conversion into HPA. Combining both stereoselective serine‐TAs and transketolase using the inexpensive racemic D/L‐serine led to high Ery yield (82%).

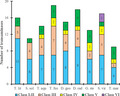

## Introduction

Transaminases (TAs, EC 2.6.1.X) are a widely distributed group of enzymes that play an important role in cell metabolism specifically in the nitrogen recycling and fixation from internal and exogenous amino acids. TAs are pyridoxal‐5′‐phosphate (PLP)‐dependent enzymes whose ping–pong mechanism reaction is very well understood (Ward and Wohlgemuth, 2010). TAs catalyse the transfer of an amine group from an amine donor to an amine acceptor (ketone or aldehyde) yielding the corresponding ketone or aldehyde and a new chiral amine compound, respectively, where the cofactor PLP is fully regenerated at the end of the reaction (Eliot and Kirsch, 2004).

Based upon protein sequence homology analyses, TAs have been divided into six protein families (P*fam*) or classes (Mehta and Hale, 1993; Hwang *et al*., 2005; Steffen‐Munsberg *et al*., 2015) as follows: I–II (P*fam* 00155, e.g. AspTA and aromatic TAs); III or also called ω‐TAs (P*fam* 00202, e.g. amine TAs and ornithine TA), which have broad substrate specificities being useful for the synthesis of chiral amines and unnatural amino acids (Kelly *et al*., 2018); IV (P*fam* 01063, e.g. branched‐chain TAs and D‐amino acid TAs); V (P*fam* 00266, e.g. PSer TA) and VI (P*fam* 01041) also known as sugar TAs, mostly involved in the amino sugar synthesis occurring in cells producing secondary metabolites (Nedal and Zotchev, 2004). The sequence similarities between TAs appear to be closely related with their functional specificity, and in spite of the TAs varied amino donor specificity, the common amino acceptor within cells is either α‐ketoglutarate or pyruvate (Mehta *et al*., 1993); this is particularly interesting as these two compounds can be used for initial TAs activity screening and substrate scope studies.

TAs from all classes have been utilized in the synthesis of fine chemicals and pharmaceuticals (Savile *et al*., 2010; Kelly *et al*., 2020), particularly for the sustainable production or chiral amine compounds such as α‐, β‐ and ω‐amino acids, unnatural amino acids and primary amines (aliphatic, aromatic and aminoalcohols) (Cárdenas‐Fernández et al., 2012a,b; Rudat and Brucher, 2012; Guo and Berglund, 2017). It is estimated that 40% of pharmaceuticals contain a chiral amine component in their structure, however, their chemical synthesis is still challenging (Ghislieri and Turner, 2014). The main advantages of TAs application compared with chemical synthesis are their excellent regio‐ and stereoselectivity as well as acting under mild reaction conditions avoiding the utilization of often toxic catalysts and solvents. Several review papers have been published summarizing the great potential of TAs and their current applications at industrial scale, as well as highlighting the importance to keep searching for novel TAs with better features than the existing ones (Kohls and Steffen‐Munsberg, 2014; Guo and Berglund, 2017; Kelly *et al*., 2020).

Thermostable enzymes possess high optimum temperatures and show extended activity half‐lives at high temperatures, being more robust and stable in such conditions and able to retain their activity in the presence of protein denaturing organic solvents, which are often needed for solubilizing hydrophobic substrates compared with their mesophilic counterparts (Atalah *et al*., 2019). Performing biocatalytic processes at high temperatures allows increasing substrate and product solubility as well as minimizing the risk of microbial contamination (Littlechild *et al*., 2007; Mehta *et al*., 2016). For all these reasons, thermostable enzymes are highly desirable as biocatalysts. Currently, a few thermostable enzymes are used in combination with conventional chemical synthesis for the production of pure drugs of interest to pharmaceutical and agrochemical companies (Littlechild, 2015).

Thermophilic microorganisms, including bacteria and archaea that live in natural high‐temperature environments, are an important source of thermostable enzymes (Vieille and Zeikus, 2001; Littlechild, 2015). Nowadays, most of the TAs described are from mesophilic origin, isolated mainly from single strains and more recently from metagenomes (Baud *et al*., 2017; Leipold *et al*., 2019). Only very few thermostable TAs have been reported to date with different applications (Chen *et al*., 2016; Mathew *et al*., 2016; Ferrandi *et al*., 2017; Bawn *et al*., 2018; Márquez and Atalah, 2019); however, thermostable TAs are not currently commercially available (Atalah *et al*., 2019). The discovery of novel thermophilic TAs remains rather under‐explored despite the increasing demand for these enzyme classes. The advantage of using thermostable TAs in chiral amine synthesis relies on their better compatibility with industrial requirements (as explained above) allowing the achievement of higher reaction throughputs, increased productivity and the lowering of process costs.

The objectives of this work were to clone novel putative TAs from thermophilic strains using a genome mining approach, to establish a methodology for quick enzyme expression and recovery and to confirm TAs activity via activity assays with natural amino donors–acceptors. In addition, we aim to demonstrate the applicability on this enzyme discovery process in the synthesis of hydroxypyruvate (an important chemical intermediate for biologically active chiral aminocyclitols) catalysed by stereoselective and non‐stereoselective thermostable serine‐TAs.

## Results and discussion

### Discovery and phylogeny of TAs from thermophiles

Only a few thermostable TAs have been reported to date in spite of their high demand in industrial processes for the synthesis of chiral amines used, for example, in the production of active pharmaceutical ingredients (APIs). Several approaches are taken for novel enzyme discovery including activity‐guided (using media enriched with a desired substrate) genome mining through protein sequence homology from databases, motif‐based analyses and metagenomics; each strategy having their own benefits and drawbacks.

In this work, TAs from nine extremophilic microorganism from our own collection were sought by mining for TAs in their genome sequences using their translated genomes in the NCBI or UniProtKB protein database. This *in silico* approach of using enzyme name (‘transaminase’ or ‘aminotransferase’) searches in a thermophiles’ genomes improves the chances to reveal functional thermostable TAs. Two of the selected strains (*T*. *litoralis* and *S*. *solfataricus*) are from the Archaeal kingdom; *T. aquaticus, D. geothermalis*, *D. radiodurans* and *T. maritim*a are gram‐negative bacteria, whilst *T. fusca*, *S. viridis* and *G. stearothermophilus* are gram‐positive ones.

In total 157 putative TA sequences were retrieved and then subjected to PHMMER analysis in order to predict and confirm their P*fam* annotation. Eighty‐four per cent of the TA sequences found in the database were confirmed to belong to the TAs superfamily, whilst the rest were wrongly annotated being associated with other PLP‐dependent enzyme families.

The percentage of open reading frames (ORFs) for these TAs was *T. litoralis* 0.689%, *S*. *solfataricus* 0.424%, *T. aquaticus* 0.633%, *T. fusca* 0.620%, *D. geothermalis* 0.495%, *D. radiodurans* 0.588%, *G. stearothermophilus* 0.483%, *S. viridis* 0.446% and *T. maritima* 0.487%.

**Fig. 1:**
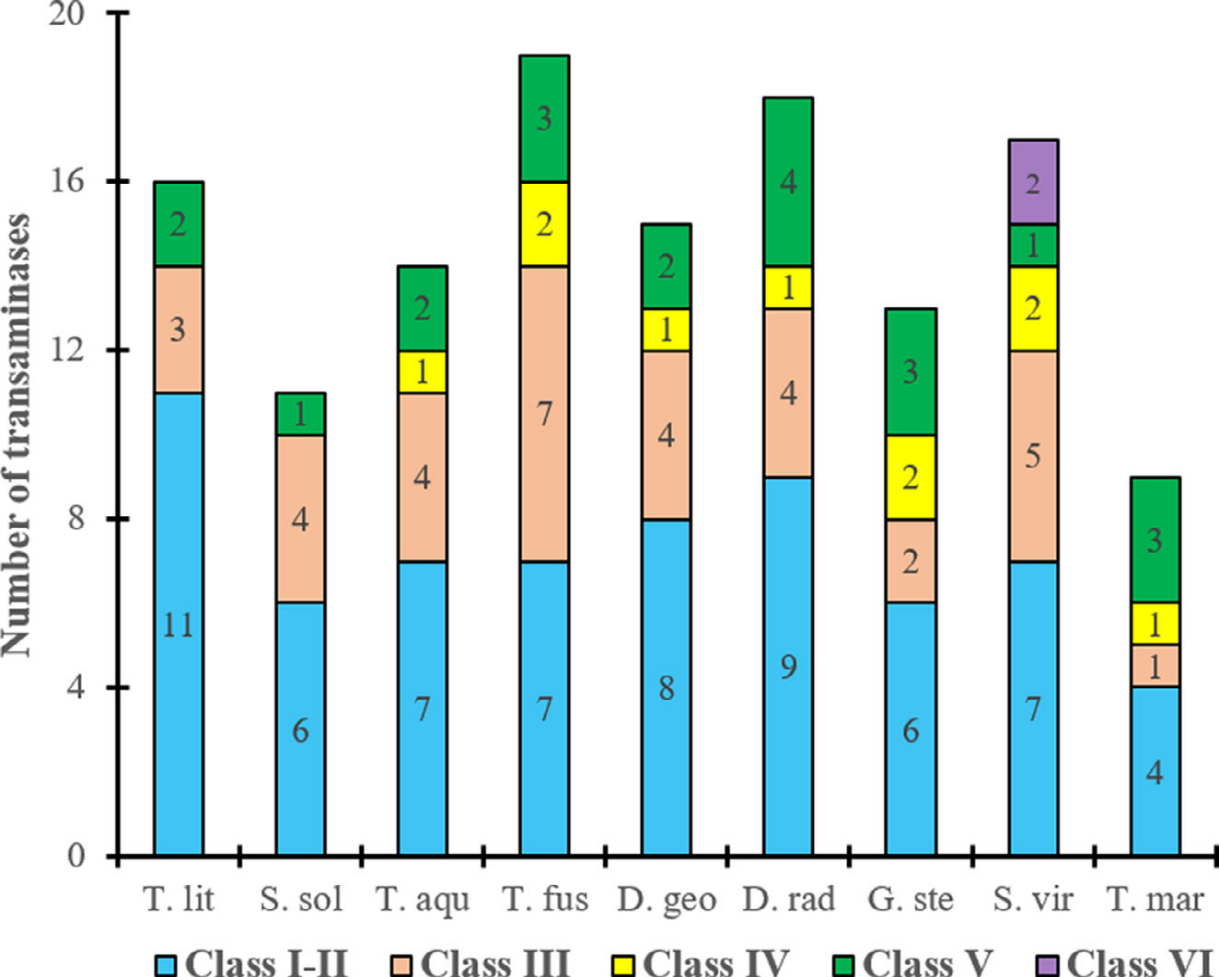
Distribution of TAs in thermophilic microorganisms. T. lit, *Thermococcus litoralis*; S. sol, *Sulfolobus solfataricus*; T. aqu, *Thermus aquaticus*; T. fus, *Thermobifida fusca* DSM 43792; D. geo, *Deinococcus geothermalis* DSM 11300; D. rad, *Deinococcus radiodurans* DSM 20539; G. ste, *Geobacillus stearothermophilus* DSM 13240; S. vir, *Saccharomonospora viridis* DSM 43017 and T. mar, *Thermotoga maritima* DSM 3109.

Distribution of TAs class per microorganism is depicted in Fig. 1. On average TAs class I–II are the most abundant one (50%), followed by type III (26%), V (16%) and VI (1%). Only 8% of the TAs are class IV, and interestingly, this type of TAs were not found in the archaea strains. Only two TAs class VI were found and these were both in the *S. viridis* strain, which was expected as this type of TAs are more commonly found in actinomycetes which are producers of secondary metabolites such as antibiotics or amino sugars. This was not the case of *T. fusca* which is also an actinomycete but it has a relatively small genome for the actinobacteria and has only one secondary metabolite gene cluster (Dimise and Widboom, 2008).

From the 132 identified TAs, 18 were previously published (Bommer, 2007; Bommer and Ward, 2013; Villegas‐Torres *et al*., 2015; Bawn *et al*., 2018), and therefore discarded from this study. TAs class VI were also not considered in this work. The rest were amplified by PCR and then cloned using our in‐house one‐pot restriction‐ligation reaction method with Type IIS endonucleases and modified pET28/29a+ plasmids (Dobrijevic *et al*., 2020). Positive clones were then confirmed by sequencing. A total of 94 putative TAs were selected to build our TAs panel in a 96‐well plate format and pQR numbers (the Ward group plasmid identifier) were consecutively assigned from pQR2501 to pQR2594 (See protein IDs in Table S1). Overall, this efficient targeted approach allowed identifying and cloning putative TAs from thermophiles with 84% success rate.

**Fig. 2:**
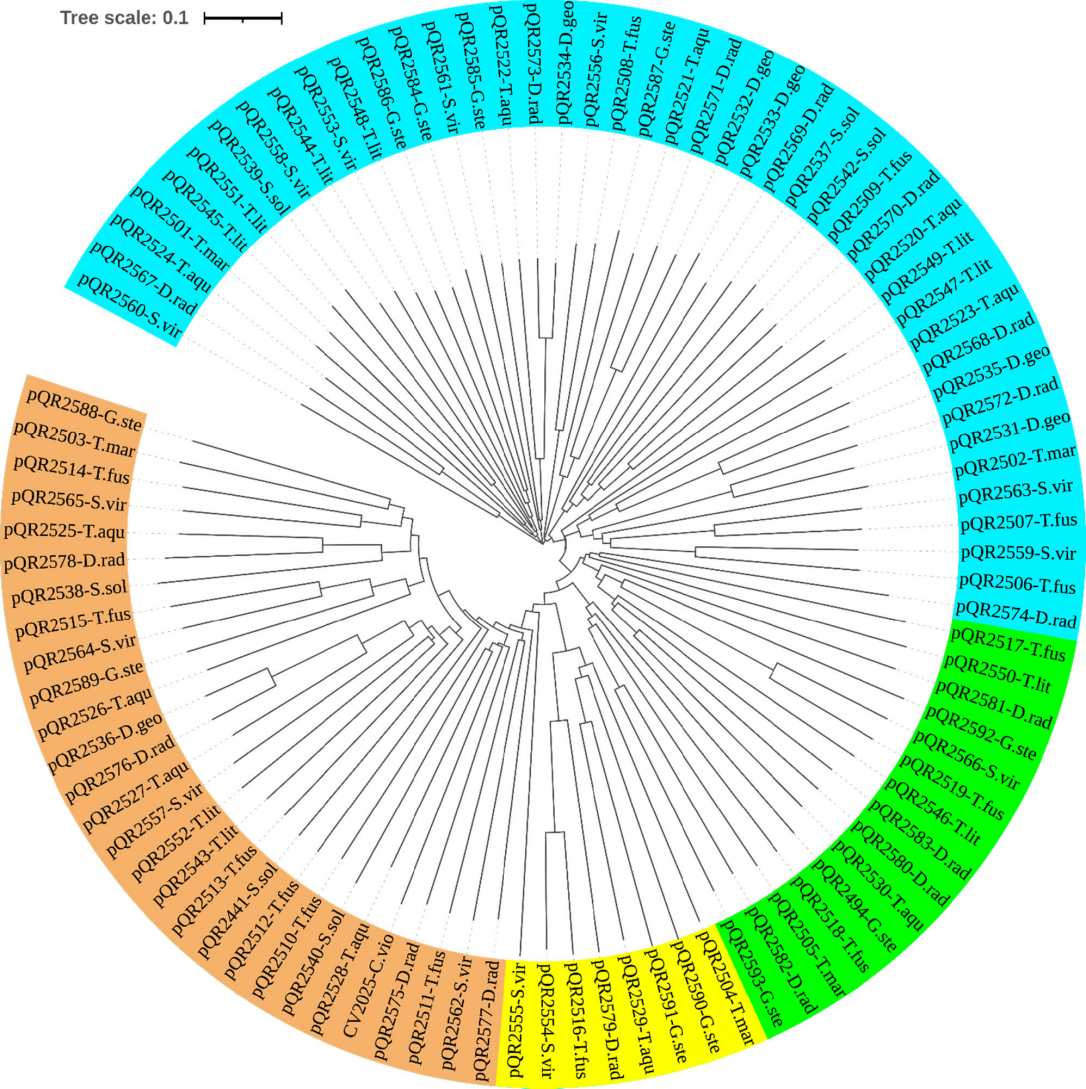
Phylogenetic tree of 94 thermophilic TAs pQR2501 to pQR2594. T. lit, *Thermococcus litoralis*; S. sol, *Sulfolobus solfataricus*; T. aqu, *Thermus aquaticus*; T. fus, *Thermobifida fusca* DSM 43792; D. geo, *Deinococcus geothermalis* DSM 11300; D. rad, *Deinococcus radiodurans* DSM 20539; G. ste, *Geobacillus stearothermophilus* DSM 13240; S. vir, *Saccharomonospora viridis* DSM 43017, T. mar, *Thermotoga maritima* DSM 3109, CV2025‐C.vio (ω‐TA from *Chromobacterium violaceum*). Multiple sequence alignment and construction of phylogenetic tree were carried out using Clustal Omega and then formatted using the iTOL server (itol.embl.de/index.shtml). Colour clades correspond to a TA class: Sky blue, TA class I–II; Orange, TA class III; Yellow, TA class IV and Green, TA class V. The species are shown and the pQR plasmid clone number corresponds to the cloned protein sequence in Table S1.

The 94 selected TAs from thermophiles and CV2025 [one of the most characterized and widely utilized ω‐TAs with proven applicability in the synthesis of chiral amines (Kaulmann *et al*., 2007)] were subjected to multiple protein sequence alignment and a phylogenetic tree was built as shown in Fig. 2. TAs were easily grouped into four clades corresponding to each TA class (I–II to V). pQR2574 is annotated as a TA class I–II but it seems to be more evolutionary related to TAs class V forming a sub‐clade together with pQR2517 and 2550. Likewise, although pQR2555 was predicted to belong to TAs class IV, it might be more closely associated with TAs class III.

Lysine residue (Lys288 in CV2025) in the active site is highly conserved among most PLP‐dependent enzymes, whose ε‐amine forms a Schiff’s base linkage with the C4‐aldehyde group of PLP, playing an important role in the biocatalytic reaction (Steffen‐Munsberg *et al*., 2015). The multiple sequence analysis revealed that 84% of the TAs showed a conserved Lys288 in their structures except for pQR2549 (class I–II), pQR2550 (class V), TAs class IV and the sub‐clade TA class V (pQR2594, 2518, 2505, 2582 and 2593); however, the latter have a conserved Lys two positions forward. In addition, Asp259 in CV2025 (responsible for the stabilization of the proton from the nitrogen of PLP pyridine ring by hydrogen bond coordination) is also highly conserved in 91% of our TAs, apart from the TAs class IV and pQR2549 (Supporting Information).

Highly conserved amino acid residues (apart from Lys and Asp in the active site) are found in most of our TAs class I–II (based on pQR2502 sequence): Gly84, Phe/Tyr/Trp106, Pro150, Gly/Ala212 and Arg315 (Supporting Information).

TAs class III are the most studied ones due to their wide variety of substrate acceptance and with an extensive application in industry (Kelly *et al*., 2018). An intra‐protein sequence alignment of TAs class III in our panel (Supporting Information) showed low homology (23–30%) related to CV2025. Likewise, 14 amino acid residues are highly conserved including key amino acid residues in the active site associated with the PLP biding site (Humble *et al*., 2012; Henríquez and Cardós‐Elena, 2020) based on CV2025 sequence: Glu226, Asp259, Arg267, Lys288, Thr321 and Arg374 (except pQR2511); in addition, Pro426 was also highly conserved. This shows the potential that these TAs might possess with improved features in terms of thermostability and substrate scope.

From the protein sequence point of view, TAs class IV seem to distinguish themselves the most from the other TAs. Only four amino acids are highly conserved (based on pQR2590 sequence, TA further studied here): Arg54, Glu181, Asn186 and Leu205; whilst their Lys288 equivalent is either Cys (for sub‐clade pQR2516, 2554, 2555 and 2579) or Thr (for sub‐clade pQR2504, 2529, 2590 and 2591), being presumably an indicative of substrate specificity, not further studied here.

Regarding the 15 TAs class V, they have a low homology between 12% and 38% to each other (except pQR2519 and 2566 with 69% homology) and five amino acids are highly conserved (based on pQR2519, TAs further studied here): Gly152, Asp172, Asp188 and Lys196 and Arg346.

A further intra‐ and inter‐protein sequence analysis would be necessary to better understand TAs phylogeny; however, this is outside the scope of this study.

In addition, the average molecular weight of selected TAs is 42 kDa ranging between 17 (pQR2549) and 57 kDa (pQR2593), 88% of them have a molecular weight between 35 and 48 kDa. See Supporting Information for more details regarding some of the selected TAs features including protein accession numbers.

### Expression and activity profile of TAs from thermophiles

Enzyme expression and recovery of all 94 TAs at a time in a 96‐deep‐well plate format was initially performed with LB media, then cells were lysed in a water bath sonicator, allowing the recovery of active TAs in one go, avoiding the addition of chemical lysis buffers that might lead to enzyme inactivation and false negatives during activity screening. Low or poor expression was observed in the soluble fraction for some following SDS‐PAGE, which can be as a result of low cell density at the end of cultivation, the expression host used (associated with codon bias or inclusion body formation) and/or cultivation conditions (Rosano and Ceccarelli, 2014). Different strategies to improve protein expression including the use of enriched media (e.g. Terrific broth) and chemical chaperones are suggested prior to changing expression host or synthesizing codon‐optimized genes that can be more laborious and expensive, especially when working with a large number of enzymes. Addition of chemical chaperones such as amino acids, sugars (e.g. d‐sorbitol) or chaotropes in the cultivation medium have proven to be beneficial to produce recombinant enzymes in their soluble form as they inhibit or diminish the formation of inclusion bodies or protein aggregates when using *E. coli* for protein expression (Prasad and Khadatare, 2011; Yang and Zhang, 2013). A combination of different cultivation conditions using enriched cultivation media with D‐sorbitol as chemical chaperone as well as by lowering the temperature after induction was tested. Based upon SDS‐PAGE analysis of the clear lysates only (Supporting Information), we concluded that the best cultivation conditions were as follows: Terrific broth, D‐sorbitol 0.5 M, PLP 0.2 mM and phosphate buffer 0.1 M pH 7 and 20°C post‐induction. There was an improvement on the TAs expression in most of the cases based on the SDS‐PAGE analysis. In the chemical chaperon media, 59% of TAs were well expressed and 17% showed low expression level, whilst 24% exhibited poor or not apparent expression (Table 1).

**Table 1:**
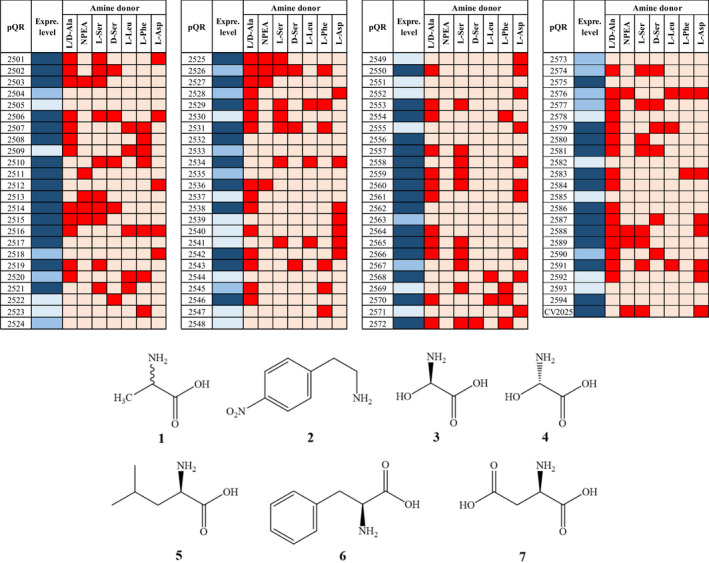
TAs panel expression and qualitative activity screening.

Enzyme expression level using chemical chaperon media was verified on SDS‐PAGE: 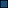 – good expression; 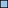 – low or poor expression and 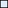 – no apparent expression. Qualitative activity screening was carried out in duplicate (two independent experiments): 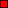 – positive activity and 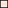 – no activity detected). Amino donor substrates (with numbered chemical structures): ***1***, l/d‐alanine; ***2***, 2‐(4‐nitrophenyl)ethan‐1‐amine (NPEA); ***3***, l‐serine; ***4***, d‐serine; ***5***, l‐leucine; ***6***, l‐phenylalanine; ***7***, l‐aspartate. The acceptor was pyruvate for the ω‐TA and α‐KG for the other TAs.

Then, in order to verify TA activity, initial qualitative high‐throughput screening assays with a series of amino donors substrates and either pyruvate or KG as amino acceptors were tested. As shown in Table 1, 80% of TAs showed at least 1 type of activity; from these, 32 were able to accept more than two substrates, eight TAs accepted four substrates and two TAs (pQR2516 and 2576 in panel positions C2 and G4, TAs class IV and III, respectively) accepted five out of the seven substrates tested. TAs class III are probably the most interesting ones from the application point of view; herein, 44% of the TAs class III showed some sort of activity, these being the only ones able to accept 2‐(4‐nitrophenyl)ethan‐1‐amine, an specific substrate for ω‐TA activity. In addition, 16 TAs that were not apparently expressed showed activity, which can be interpreted as TAs with high specific activity; whilst 12 well‐expressed enzymes showed no activity probably due to the lack of appropriate substrates.

### Novel serine‐TAs from thermophiles for HPA synthesis

Hydroxypyruvate is an expensive but vital substrate for C‐C bond forming enzymes such as transketolases when used in biocatalysis, being a chemical intermediate for the synthesis of monosaccharides and aminocyclitols. Many strategies have been studied for its *in‐situ* production in cascade reactions focusing on enzymatic methods using either TAs (Villegas‐Torres *et al*., 2015; Lorillière *et al*., 2017; Bawn *et al*., 2018) or d‐amino acid oxidases (L'enfant *et al*., 2019) with either L‐serine or d‐serine as precursors where the TA removes the amine moiety to make hydroxypyruvate *in situ*. Despite the progress achieved regarding the HPA enzymatic synthesis, the price of pure l‐serine or d‐serine remains high. Hence, we aimed first to seek non‐stereoselective serine TAs, as the racemic mixture l/d‐serine is on average up to eight times cheaper than the pure stereoisomers.

**Fig. 3:**
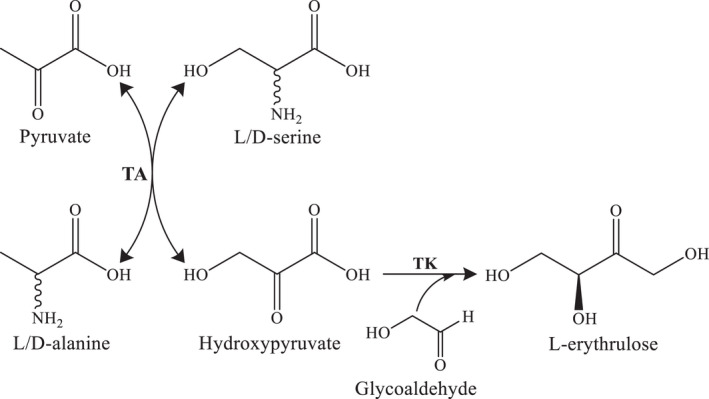
Cascade reaction scheme for the synthesis of hydroxypyruvate and L‐erythrulose catalysed by serine transaminase (TA) and transketolase (TK).

Based upon Abs_600nm_ for the WST‐1 assay of our TAs from thermophiles panel, 18 and 17 TAs were able to accept either l‐ or d‐serine, respectively; from these ones, eight TAs accepted both isomers almost equally (based on l‐ and d‐serine Abs_600nm_ ratio) considered then as non‐stereoselective serine‐TAs. TAs pQR2502 (*T. maritima*), pQR2526 (*T. aquaticus*), pQR2577 and 2581 (both from *D. radiodurans*) (Table 2) were selected for the synthesis of HPA from L/D‐serine (Fig. 3). The two chosen had the least cross reactivity to the other amino acid besides l‐alanine which is almost always an activity with transaminases and one that may be desired in using pyruvate as the acceptor in some cascades. All bioconversion reactions were carried out at 40°C to avoid HPA degradation, as this compound is unstable at temperature above 50°C (Lorillière *et al*., 2017) and if it is desired that the HPA build up as the end‐product, it needs to be under conditions where it is stable. However, if the HPA is removed using a second enzyme such as a transketolase (TK), then the coupled cascade of TA and TK can be carried out at high temperatures of 70 or 80°C because the HPA generated is immediately used by the TK. HPA concentration was determined by HPLC analysis. As shown in Table 2, non‐stereoselective serine‐TA activity was confirmed when testing both serine isomers separately, showing in all the cases relatively low HPA yields. When bioconversion reactions were performed with racemic l/d‐serine, reaction yields were even lower than the yields showed for the single isomer, probably due to a substrate inhibition.

**Table 2:** HPA and Ery synthesis catalysed by serine‐TAs and serine‐TAs+TK respectively.

TA			HPA yield (%)			Ery yield (%)
pQR	Source	Class	l‐serine	d‐serine	l/d‐serine	l/d‐serine and TK
Non‐stereoselective serine‐TAs						
2502	*Thermotoga maritima*	I‐II	4.6 ± 0.1	8.5 ± 0.2	2.5 ± 0.1	13.4 ± 0.4
2526	*Thermus aquaticus*	III	17.5 ± 1.1	6.8 ± 0.7	8.6 ± 0.3	12 ± 0.4
2577	*Deinococcus radiodurans*	III	6.5 ± 0.2	7.3 ± 0.8	2.5 ± 0.3	N.D
2581		V	30.2 ± 0.5	1.1 ± 0.03	21.5 ± 0.4	34.5 ± 0.7
Stereoselective serine‐TAs						
2519	*Thermobifida fusca*	V	14.2 ± 1.7	N.A	5.9 ± 0.9	64.3 ± 0.7
2590	*Geobacillus stearothermophilus*	IV	N.A	33.8 ± 1.6	19.3 ± 1.3	76.9 ± 0.1
2519 + 2590			N.D	N.D	26.1 ± 0.3	82.3 ± 1.9

Ery, L‐erythrulose; HPA, hydroxypyruvic acid; N.A, no activity detected; N.D, no determined; TK, transketolase from *Thermotoga maritima*.

All reactions were carried out as in Section 2.5, with 0.5 ml reaction volume, 10 mM pyruvate, 10 mM of either l‐, d‐ or racemic l/d‐serine, at 40°C and 700 rpm for up to 24 h. All reactions were done in duplicate (from independent experiments).

The transamination reaction is reversible and suffers from unfavourable reaction equilibrium that hamper bioconversion to achieve high yields. The equilibrium can be shifted towards product formation by adding an excess of amine donor or removing the product *in situ* by coupling it to other enzymatic step (Tufvesson *et al*., 2011). Non‐stereoselective serine‐TA reactions were then coupled to a hyperthermophilic transketolase from *Thermotoga maritima* (TK_tmar_) reaction that catalyses the C‐C bond formation between HPA and glycoaldehyde to yield Ery (Fig. 3) with almost 100% conversion (Cárdenas‐Fernández *et al*., 2021). No HPA was detected in these reactions as it was immediately used by the TK_tmar_ for Ery formation. A significant improvement on transamination reaction was observed (Table 2). However, reactions yields are still low to be considered for further applications at this initial stage.

A second strategy to produce HPA from l/d‐serine was taken by combining two stereoselective serine‐TAs, one selective for L‐ and one selective for D‐serine. The WST‐1 screening assay showed that TA pQR2519 (TA class V, *T*. *fusca*) and pQR2590 (TA class IV, *G*. *stearothermophilus*) accepted solely L‐ and D‐serine respectively. Then, their substrate selectivity was confirmed by HPLC reaching HPA yields of 14.2% and 33.8% after 24 h reaction, respectively; similar conversion levels have been reported previously by using mesophilic and thermophilic serine‐TAs (Villegas‐Torres *et al*., 2015; Bawn *et al*., 2018). A potential enzyme inhibition by the isomer counterpart was observed as the HPA yields decreased by 2.4‐ and 1.8‐fold, when using serine racemic mixture. However, when the single TA reactions were coupled to TK and in presence of both isomers, the HPA yields rose back to 69.3% and 76.9%. Then, both stereoselective serine‐TAs were mixed in bioconversion reaction with the racemic l/d‐serine, achieving 26.1% HPA yield. The l/d‐serine conversion was significantly increased by 3.2‐fold when coupling the TK reaction reaching 82.3% Ery yield. An optimized cascade reaction with a L‐serine‐TA from *D. geothermalis* (Dgeo_0713) and a TK from *E. coli* was reported with almost 100% Ery yield but using threefold l‐serine excess compared with pyruvate and glycoaldehyde (Villegas‐Torres and Ward, 2018).

### Thermal characterization of TAs pQR2519 and pQR2590

**Fig. 4:**
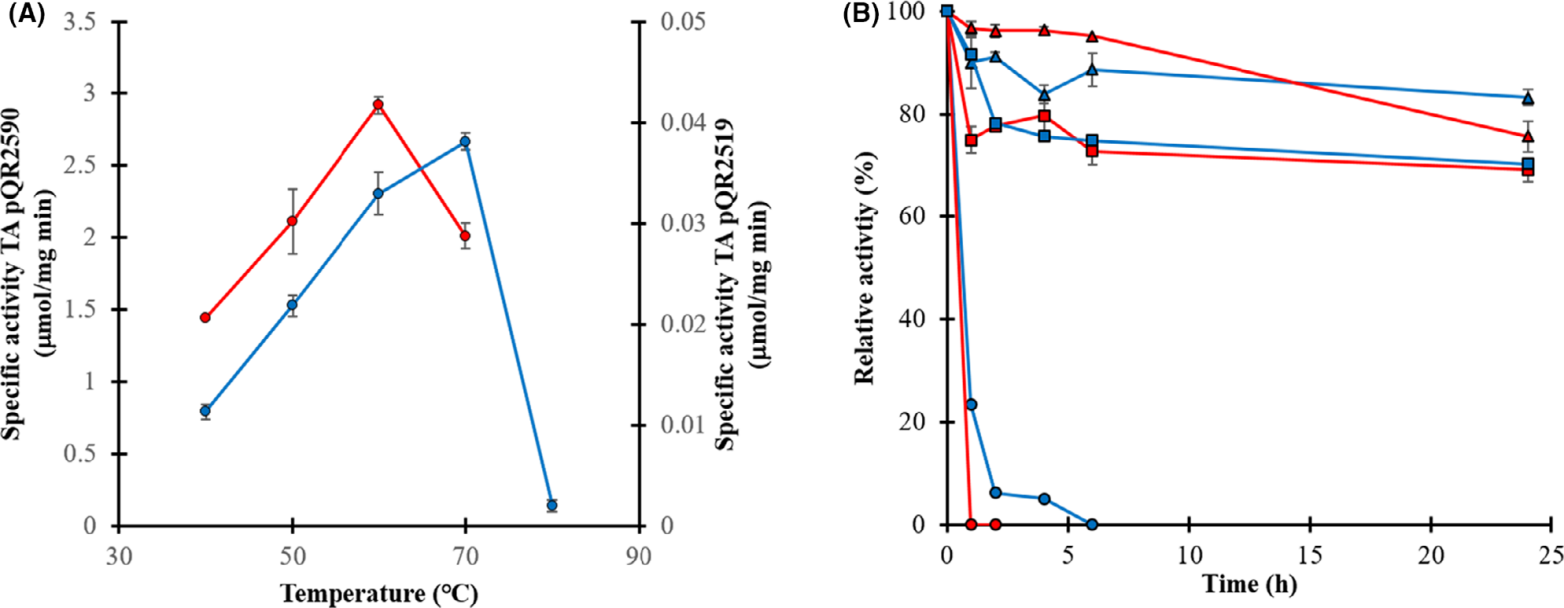
Thermal characterization of stereoselective serine‐TAs. A. Optimum temperature: TA pQR2519 (blue line) and TA pQR2590 (red line). B. Thermostability: pQR2519 (blue line), TA pQR2590 (red line), (▲) 50°C, (■) 60°C and (●) 70°C. All experiments were carried out as in Section 2.6. All reactions for initial rate calculations were done in duplicates (from independent experiments).

Stereoselective serine‐TAs pQR2519 and pQR2590 were selected for their partial characterization based on their good reaction yield performance for the bioconversion of l‐ or d‐serine into HPA (section 3.3) in order to confirm they were thermophilic enzymes. The TAs pQR2519 and pQR2590 optimum temperatures were 70 and 60°C respectively. TA pQR2590 showed a much higher specific activity towards its respective serine isomer than TA pQR2519 by two orders of magnitude (Fig. 4A). Similar specific activity values (around 3.5 µmol mg^‐1^ min^‐1^) to TA pQR2590, for the same reaction and similar reaction conditions, were reported for two thermophilic L‐serine‐TAs from *D. geothermalis* and *G. stearothermophilus* (Bawn *et al*., 2018).

The application of thermophilic enzymes is not only limited to biocatalytic reactions at high temperature (e.g. > 60°C) but they can also be used at moderate high temperatures (e.g. 40 or 50°C as in this work) showing extended half‐lives (Fig. 4B) compared with their mesophilic counterparts whose half‐lives can be only of a few minutes or hours when working at such temperatures, as it has been already shown for mesophilic TAs from *V. fluvialis* (Deepankumar *et al*., 2014)*, Rhizobium sp*. (Tang *et al*., 2020) and *B. altitudinis* (Xie *et al*., 2020). In this study, both enzymes showed to be thermostable keeping around the 80% of their initial activity after 24 h incubation at 50 and 60°C. Both serine‐TAs were fully deactivated at 70°C after 1 and 6 h for pQR2590 and pQR2519 respectively (Fig. 4B).

A particular attention is put on the thermophilic TA pQR2590, which showed a poor expression (Fig. 2) but a high specific activity. Based upon sequence homology analysis, pQR2590 belongs to TA class IV (branched‐chain amino acid transaminase) and sub‐classified as a D‐amino acid transaminase (D‐AAT, EC 2.6.1.21). D‐AATs are key enzymes in the synthesis of D‐amino acids constituent of the cell wall in gram‐positive bacteria (Kobayashi, 2019). D‐AAT are highly specific towards D‐amino acids, which is in agreement with our results as TA pQR2590 only accepted D‐Ser and D‐Ala (from racemic mixture). Therefore, TA pQR2590 has a great potential for the synthesis of unnatural D‐amino acids which have a wide application in pharmaceutical, food and cosmetic sectors (Gao and Ma, 2015).

## Conclusions

This work shows a quick and reliable methodology for the discovery, cloning, expression and screening of a high number of TAs from thermophiles. Although low expression was observed in some cases, activity was still detected during screening, suggesting high specific activity. In such cases, codon optimization is recommended for selected enzymes in order to improve expression levels for further applications.

Both strategies taken in this study for the synthesis of HPA from the inexpensive racemic l/d‐serine catalysed by thermophilic TAs are promising. However, further improvement of non‐stereoselective serine‐TAs by enzyme rational design and directed evolution would be needed in order to improve reaction yields and avoid substrate inhibition issues. The best enzymatic reaction performance was found when combining two stereoselective serine‐TAs of opposite enantioselectivity, a transketolase and racemic l/d‐serine as starting material. This multienzimatic one‐pot cascade reaction led to a significant increase in serine conversion. Co‐expression of both stereoselective serine‐TAs would contribute to reducing the process cost for these enzymes production.

## Experimental procedures

### Chemicals, strains and plasmids

All chemicals were purchased from Merck KGaA (Sigma‐Aldrich, Poole, Dorset, UK), and molecular biology reagents were from New England BioLabs, unless otherwise stated. Thermophilic strains used in this study were as follows: *Thermococcus litoralis*, *Sulfolobus solfataricus*, *Thermus aquaticus* (these three from our own collection), *Thermobifida fusca* DSM 43792, *Deinococcus geothermalis* DSM 11300, *Deinococcus radiodurans* DSM 20539, *Geobacillus stearothermophilus* DSM 13240, S*accharomonospora viridis* DSM 43017 and *Thermotoga maritim*a DSM 3109. In house‐modified plasmids pET‐28a+ and pET‐29a+ with *Sap*I or *Bsa*I restriction sites, respectively, both plasmids include *SacB* and *KanR* genes for positive selection (Dobrijevic *et al*., 2020).

### Transaminase (TA) genes mining and cloning

Putative TA protein and gene sequences were identified from the respective thermophilic microorganisms from NCBI and UniProtKB databases following an annotation search strategy using ‘transaminase’ or ‘aminotransferase’ as keywords. TAs P*fam* were then predicted with PHMMER and TA family class was assigned. Multiple sequence alignment and phylogenetic analysis of selected TAs were carried out using Clustal Omega and Jalview 2.11.1.3. The phylogenetic tree was formatted using the iTOL server (itol.embl.de/index.shtml). PCR primers for the identified TAs were designed with a *Tm* between 70 and 73°C and adding overhangs homologous to the modified pET vectors for SapI (F: 5’‐AAAGCTCTTCGATG‐3’ and R: 5’‐AAAGCTCTTCGGTG‐3’) and BsaI (F: 5’‐AAAGGTCTCTTATG‐3’ and R: 5’‐AAAGGTCTCGGGTG‐3’). TAs genes were amplified using Phusion^®^ High‐Fidelity PCR Master Mix with GC Buffer, 100 ng of genomic DNA or alternatively 2 µl of strain glycerol stock, primers concentration 0.5 µM and DMSO 5% in 50 µl reaction. Two‐step PCRs were carried out as follow: initial denaturation, 98°C for 3 min; 30 cycles of denaturation at 98°C for 10 s; annealing–extension at 72°C for 30 s and final extension, 72°C for 10 min. Amplicons were confirmed in agarose gel electrophoresis (1% in TBE buffer) and PCR products were then recovered from the gel following Monarch^®^ DNA Gel Extraction Kit protocol. TAs cloning was then performed with a one‐pot restriction–ligation reaction method with either SapI or BsaI and the modified pET28a+ and pET29a+ vectors, respectively (Dobrijevic *et al*., 2020), and transformed into chemically competent *E. coli* NovaBlue and grown overnight at 37°C in LB agar with kanamycin 50 μg ml^−1^ and sucrose 10%. Colonies were picked and then grown overnight in 10 ml of LB broth with kanamycin 50 μg ml^−1^ at 37°C and 250 rpm, and plasmids were extracted using QIAprep Spin Miniprep kit (Qiagen) and sent for sequencing (Eurofins Genomics) to confirm gene sequences. Positive plasmid constructs were then transformed into an expression host either *E. coli* BL21 (DE3) or *E. coli* Rosetta 2 (DE3) (Supporting Information). All recombinant TAs have a C‐terminal His_6_Tag.

### Enzymes expression and cell lysis

Ninety‐four TAs were selected and put in a 96‐well plate format (position from A1 to H10) including the ω‐TA from *Chromobacterium violaceum* (CV2025) as positive control (H11) and *E. coli* BL21 (DE3) with empty pET29a+ as negative control (H12). TAs were initially expressed in a 96‐deep‐well plate with 1 ml of LB broth per well with kanamycin 50 μg ml^−1^ at 37°C and 1200 rpm in Thermomixer™ C (Eppendorf UK Limited, Arlington Business Park, Stevenage, UK), grown until OD_600nm_ ~ 0.6 and then induced with isopropyl β‐d‐1‐thiogalactopyranoside (IPTG) 0.1 mM final concentration and further incubated at 25°C for 15 h. In order to enhance enzyme expression, chemical chaperone media (1 ml per well) were used containing: Terrific broth, D‐sorbitol (0.5 or 0.4 M), sodium phosphate buffer (100 or 50 mM) pH 7, PLP 0.2 mM and kanamycin 50 μg ml^−1^ at 37°C and 1200 rpm; IPTG induction (0.5 mM final concentration) was done at OD_600nm_ ~ 1.5 and temperature was then reduced (25 or 20°C) for 15 h. TA plate was then centrifuged (4500 rpm at 4°C for 30 min) and cell pellets were re‐suspended in 0.2 ml of 50 mM TRIS‐HCl buffer pH 7 containing PLP 0.2 mM. Cells were disrupted by partially submerging the TA plate in a water bath sonicator (Bransonic 2800 CPX2800H‐E, Branson) at maximum power for 20 min at 10°C, and the plate was rotated clockwise every 5 min. Then, the TA plate was centrifuged as above. The clarified lysates were recovered in a fresh 96‐well plate and kept at 4°C for enzymatic activity screening and protein quantification. Enzyme expression was confirmed with SDS‐PAGE analysis with Novex^®^ TBE 10% gels (Invitrogen, Waltham, MA, USA) and NuPAGE™ MOPS‐SDS running buffer (Thermo Fisher Scientific, Waltham, MA, USA).

### Activity screening assay

End‐point colorimetric assays were performed for different substrates. First, alanine‐TA activity was assay with 200 mM D/L‐alanine and 2 mM α‐ketoglutarate (KG) as amino donor and acceptor, respectively, followed by the addition of 2,4‐dinitrophenyl hydrazine (1 mM in 1 M HCl) after overnight incubation, resulting in a red colouration for active enzymes (Y. Sheludko and F. Wolf‐Dieter, personal communication, 2019). Second, l‐ and d‐serine TA assay was performed following the method reported by Bommer and Ward (2016), using 10 mM l‐ or d‐serine (amine donor) and 10 mM pyruvate, incubated overnight and followed by the addition of 0.1 mg ml^‐1^ WST‐1 (BioVision) as reducing agent leading to a blue colour formation with Abs_600nm_ higher than 0.05 for positive reactions. Third, ωTA activity was confirmed with 10 mM pyruvate and 25 mM 2‐(4‐nitrophenyl)ethan‐1‐amine as amino donor, the reaction was incubated overnight and positive reactions led to a formation of an orange‐red coloration (Baud *et al*., 2015). All these end‐point assay reactions were carried out in 96‐well plates with 20 µl of clarified lysate and 180 µl of substrate reagent mixture containing 0.2 mM PLP in 50 mM TRIS‐HCl buffer pH 7, and incubated at 50°C.

Enzymatic kinetic assays for l‐leucine, l‐phenylalanine and l‐aspartate TAs as amino donors were performed following Walton *et al*.’s (2013) spectrophotometric method with KG as amine acceptor. Reaction mixture per well consists of 20 µl of clarified lysate and 180 µl of freshly prepared reagent mix (5 mM amino acid, 1 mM KG, 5 U ml^‐1^ glutamate dehydrogenase from bovine liver and 0.5 mM NAD+ in 50 mM TRIS–HCl buffer pH 7). Reactions were carried out at 40°C, increased absorbance due to NADH formation was monitored at 340 nm every 1.5 min per well for up to 2 h in a plate reader (CLARIOstar Plus, BMG Labtech). Positive reactions were considered when Δ Abs min^‐1^ of sample was 1.5‐fold greater than Δ Abs min^‐1^ of the negative control (position H12).

Final protein concentration for both end‐point colorimetric and kinetic assays ranged from 0.05 to 5.4 mg ml^‐1^.

### Synthesis of hydroxypyruvate (HPA) and l‐erythrulose (Ery)

TA‐catalysed HPA synthesis was carried out with pure non‐stereoselective serine‐TA pQR2502, pQR2526, pQR2577 and pQR2581 (plate positions A2, C2, G5 and G9, respectively); and pure stereoselective l‐serine‐TA pQR2519 (plate position B7) and d‐serine‐TA pQR2590 (plate position H6). The reaction mixture content was 10 mM of either l‐, d‐ or racemic l/d‐serine, 10 mM pyruvate, 0.2 mM PLP and 0.3 mg ml^‐1^ TA in 50 mM TRIS‐HCl buffer pH 7. In order to increase serine conversion, TA reaction was coupled to a pure transketolase from *Thermotoga maritima* (TK_tmar_) to yield Ery. Cascade reaction condition was as above with the addition of 10 mM glycoaldehyde, 0.25 mg ml^‐1^ TK_tmar_, 9 mM MgCl_2_ and 2.4 mM thiamine diphosphate. All reactions were carried out at 40°C and 500 rpm in Thermomixer^TM^ C.

### Thermal characterization of stereoselective serine‐TAs

Optimum temperature of pure stereoselective serine‐TAs was determined by measuring the initial reaction rates (for up to 15 min) of the HPA synthesis reaction (see Section 2.5) with 1 mg ml^‐1^ of TA pQR2519 and 0.1 mg ml^‐1^ of TA pQR2590, in 50 mM TRIS‐HCl buffer pH 7 with PLP 0.2 mM and incubated from 40 to 80°C with 10°C intervals in a Thermomixer™ C. Serine‐TAs thermostability was evaluated by incubating 10 and 0.5 mg ml^‐1^ of TA‐B7 and TA‐H6, respectively, at 50, 60 and 70°C in 50mM TRIS‐HCl buffer pH 7 with PLP 0.2 mM for up to 24 h. Samples were taken periodically for enzyme activity analysis calculating initial reaction rates in the HPA synthesis reaction at 60°C.

### Analytical methods and general procedures

Selected serine‐TAs were purified by IMAC methodology with 1 or 5 ml Ni‐NTA agarose columns (Qiagen). Clarified lysate was prepared and the columns were equilibrated with 10 mM imidazole, the washing step was with 50 mM imidazole and enzyme elution with 0.5 M imidazole, all imidazole solutions were prepared in 50 mM TRIS‐HCl buffer with 0.3 M NaCl adjusted to pH 7. All fractions were collected and kept for subsequent protein quantification and SDS‐PAGE analysis.

Hydroxypyruvate and Ery were analysed by HPLC (Villegas‐Torres *et al*., 2015) with a Ultimate 3000+ HPLC (Thermo Fisher Scientific) fitted with a Aminex HPX‐87H column (Bio‐Rad Laboratories Ltd., Watford, UK) and TFA 0.1% at 0.6 ml min^‐1^ for 20 min (see chromatogram in Supporting Information). Retention times were 8.5 and 11.7 min respectively. All quantitative analyses were performed measuring peak area using the external standard method.

Protein quantification was carried with Quick Start Bradford Protein Assay (Bio‐Rad) using bovine serum albumin as protein standard.

## Conflict of interest

None declared.

## Supporting information


**Table S1**. Thermophilic transaminase panel and pQR numbers (the Ward group plasmid identifier). Expression host system either *E. coli* BL21 (DE3) or (^a^) *E. coli* Rosetta 2 (DE3).
**Fig. S1**. Segment of the multiple sequence alignment of the thermophilic TA proteins, highlighting (in purple boxes) the key conserved amino acid residues Asp259 and Lys288 in the active site (reference position number for CV2025).
**Fig. S2**. Segment of the multiple sequence alignment of thermophilic TAs class I‐II, highlighting (in coloured boxes) the conserved amino acid residue (reference position number for pQR2502).
**Fig. S3**. Segment of the multiple sequence alignment of thermophilic TAs class III, highlighting (in coloured boxes) the conserved amino acid residues (reference position number for CV2025).
**Fig. S4**. Protein sequence alignment of thermophilic TAs class IV, highlighting (in coloured boxes) the conserved amino acid residues (based on position number for pQR2590). Cys/Thr243 equivalent to Lys288 (CV2025).
**Fig. S5**. Segment of the multiple sequence alignment of thermophilic TAs class V. Coloured boxed are the conserved amino acid residues (reference position for pQR2519).
**Fig. S6**. Expression of thermophilic TAs using a chemical chaperon media. Media composition: Terrific broth, D‐sorbitol 0.5 M, PLP 0.2 mM and phosphate buffer 0.1 M pH 7. Cultivation conditions: 1 mL of media per well at 1200 rpm at 37°C until OD_600nm_ ~ 1.5; then induced with IPTG 0.5 mM and temperature reduced to 20°C for additional 15 h. Label per lane represents the position in the plate panel.
**Fig. S7**. Chromatogram showing Hydroxypyruvate (HPA) and L‐erythrulose (Ery). HPLC analysis as explained in Section 2.7. Standards at 5 mM of each chemical.
