# Supplementary material for: Novel transaminases from thermophiles: from discovery to application
Source: Microb Biotechnol. 2021 Oct 29;15(1):305–17. doi: 10.1111/1751-7915.13940 (PMC8719814; doi:10.1111/1751-7915.13940)

**Novel thermophilic transaminases: from discovery to application**

**Supplementary Information**

Max Cárdenas-Fernández^a,b^, Oliver Sinclair^a^ and John M Ward^a^*.

^a^ University College London, Department of Biochemical Engineering, Gower St, WC1E 6BT, London, UK

^b^ School of Biosciences, University of Kent, Canterbury, CT2 7NJ, Kent, UK

* Corresponding author: [j.ward@ucl.ac.uk](mailto:j.ward@ucl.ac.uk), Telephone: +44 020 7679 9568

**Supplementary Table 1: Thermophilic transaminase panel and pQR numbers (the Ward group plasmid identifier).** Expression host system either *E. coli* BL21(DE3) or (^a^) *E. coli* Rosetta 2(DE3)

| **pQR** | **Plate position** | **TA class** | **Source** | **Protein Accession number** | **Amino acids content** | **Protein MW (kDa)** |
| --- | --- | --- | --- | --- | --- | --- |
| 2501 | A1 | I-II | *Thermotoga*  *maritima* | NP_229498.1 | 397 | 44.9 |
| 2502 | A2 | I-II |  | NP_228846.1 | 335 | 39.3 |
| 2503 | A3 | III |  | NP_229582.1 | 385 | 42.9 |
| 2504 | A4 | IV |  | NP_228640.1 | 273 | 31.2 |
| 2505 | A5 | V |  | NP_229172.1 | 413 | 46.7 |
| 2506 | A6 | I-II | *Thermobifida*  *fusca* | WP_016189417.1 | 361 | 39.1 |
| 2507 | A7 | I-II |  | WP_011291598.1 | 367 | 39.2 |
| 2508 | A8 | I-II |  | WP_011292676.1 | 430 | 45.6 |
| 2509 | A9 | I-II |  | WP_011292067.1 | 440 | 48.1 |
| 2510 | A10 | III |  | WP_011291133.1 | 429 | 45.6 |
| 2511 | A11 | III |  | WP_011290653.1 | 431 | 45.5 |
| 2512 | A12 | III |  | WP_011290748.1 | 421 | 45.7 |
| 2513 | B1 | III |  | WP_011291137.1 | 448 | 47.4 |
| 2514 | B2 | III |  | WP_011292477.1 | 403 | 42.1 |
| 2515 | B3 | III |  | WP_011292670.1 | 418 | 43.9 |
| 2516 | B4 | IV |  | WP_011291063.1 | 373 | 40.7 |
| 2517 | B5 | V |  | WP_011290475.1 | 368 | 38.7 |
| 2518 | B6 | V |  | WP_011293202.1 | 507 | 55.6 |
| 2519 | B7 | V |  | WP_011290693.1 | 373 | 40.1 |
| 2520 | B8 | I-II | *Thermus*  *aquaticus* | WP_003046139.1 | 396 | 43.5 |
| 2521 | B9 | I-II |  | WP_003047496.1 | 356 | 39.5 |
| 2522 | B10 | I-II |  | WP_003046746.1 | 377 | 41.4 |
| 2523 | B11 | I-II |  | WP_003048749.1 | 331 | 36.6 |
| 2524 | B12 | I-II |  | WP_003045685.1 | 372 | 41.2 |
| 2525 | C1 | III |  | WP_003043516.1 | 396 | 43.6 |
| 2526 | C2 | III |  | WP_003048230.1 | 414 | 44.1 |
| 2527 | C3 | III |  | WP_003048707.1 | 432 | 46.1 |
| 2528 | C4 | III |  | WP_003045553.1 | 438 | 48.2 |
| 2529 | C5 | IV |  | WP_003049538.1 | 318 | 35.2 |
| 2530 | C6 | V |  | WP_003045787.1 | 352 | 37.9 |
| 2531 | C7 | I-II | *Deinococcus geothermalis* | WP_011529268.1 | 363 | 39.7 |
| 2532 | C8 | I-II |  | WP_011529924.1 | 414 | 45.1 |
| 2533 | C9 | I-II |  | WP_011530559.1 | 474 | 49.6 |
| 2534 | C10 | I-II |  | WP_011530884.1 | 391 | 41.8 |
| 2535 | C11 | I-II |  | WP_011525705.1 | 286 | 30.1 |
| 2536 | C12 | III |  | WP_011530126.1 | 458 | 49.3 |
| 2537^a^ | D1 | I-II | *Sulfolobus*  *solfataricus* | WP_009988922.1 | 401 | 54.6 |
| 2538^a^ | D2 | III |  | WP_009990385.1 | 388 | 43.3 |
| 2539^a^ | D3 | I-II |  | WP_009991561.1 | 356 | 40.5 |
| 2540^a^ | D4 | III |  | WP_009988652.1 | 444 | 50.6 |
| 2541^a^ | D5 | III |  | WP_009991724.1 | 440 | 48.8 |
| 2542^a^ | D6 | I-II |  | WP_009989665.1 | 380 | 42.8 |
| 2543 | D7 | III | *Thermococcus*  *litoralis* | WP_004066380.1 | 447 | 49.6 |
| 2544^a^ | D8 | I-II |  | WP_004067977.1 | 364 | 40.9 |
| 2545^a^ | D9 | I-II |  | WP_004069622.1 | 392 | 43.9 |
| 2546 | D10 | V |  | WP_004067193.1 | 381 | 42.7 |
| 2547 | D11 | I-II |  | WP_004067152.1 | 321 | 36.6 |
| 2548 | D12 | I-II |  | WP_004066529.1 | 391 | 44.6 |
| 2549 | E1 | I-II |  | WP_020953796.1 | 154 | 17.4 |
| 2550 | E2 | V |  | WP_004067120.1 | 398 | 44.6 |
| 2551 | E3 | I-II |  | WP_004067963.1 | 375 | 42.7 |
| 2552^a^ | E4 | III |  | WP_004066800.1 | 458 | 50.2 |
| 2553 | E5 | I-II | *Saccharomonospora*  *viridis* | WP_012795871.1 | 441 | 43.8 |
| 2554 | E6 | IV |  | WP_015785427.1 | 367 | 40 |
| 2555 | E7 | IV |  | WP_015785865.1 | 273 | 29.3 |
| 2556 | E8 | I-II |  | WP_015786676.1 | 415 | 44.3 |
| 2557 | E9 | III |  | WP_015787306.1 | 419 | 44.4 |
| 2558 | E10 | I-II |  | WP_015788064.1 | 392 | 42.3 |
| 2559 | E11 | I-II |  | WP_012795703.1 | 351 | 37.7 |
| 2560^a^ | E12 | I-II |  | WP_012795756.1 | 387 | 41.7 |
| 2561 | F1 | I-II |  | WP_012796126.1 | 363 | 38.6 |
| 2562 | F2 | III |  | WP_015785448.1 | 430 | 46.5 |
| 2563 | F3 | I-II |  | WP_037308628.1 | 362 | 38.9 |
| 2564^a^ | F4 | III |  | WP_015786705.1 | 416 | 44.1 |
| 2565^a^ | F5 | III |  | WP_015786863.1 | 395 | 41.5 |
| 2566^a^ | F6 | V |  | WP_015787660.1 | 376 | 40.7 |
| 2567 | F7 | I-II | *Deinococcus radiodurans* | WP_010887316.1 | 381 | 40.6 |
| 2568 | F8 | I-II |  | WP_010883990.1 | 335 | 35.5 |
| 2569 | F9 | I-II |  | WP_010887673.1 | 378 | 41.6 |
| 2570 | F10 | I-II |  | WP_010888227.1 | 420 | 45.2 |
| 2571 | F11 | I-II |  | WP_010888092.1 | 401 | 44.7 |
| 2572 | F12 | I-II |  | WP_010889086.1 | 361 | 39.5 |
| 2573 | G1 | I-II |  | WP_010888896.1 | 382 | 40.8 |
| 2574 | G2 | I-II |  | WP_010888972.1 | 424 | 45.9 |
| 2575 | G3 | III |  | WP_010889381.1 | 430 | 44.9 |
| 2576 | G4 | III |  | WP_010889289.1 | 454 | 48.9 |
| 2577 | G5 | III |  | WP_010888054.1 | 510 | 55.8 |
| 2578 | G6 | III |  | WP_010887440.1 | 465 | 50.1 |
| 2579 | G7 | IV |  | WP_010888263.1 | 358 | 39.4 |
| 2580 | G8 | V |  | WP_010887634.1 | 391 | 41.3 |
| 2581 | G9 | V |  | WP_010889090.1 | 379 | 41 |
| 2582 | G10 | V |  | WP_081816088.1 | 479 | 52.7 |
| 2583 | G11 | V |  | WP_010887991.1 | 390 | 42.6 |
| 2584 | G12 | I-II | *Geobacillus stearothermophilus* | WP_049624554.1 | 385 | 42.5 |
| 2585 | H1 | I-II |  | WP_053532246.1 | 391 | 42.5 |
| 2586 | H2 | I-II |  | WP_033016287.1 | 390 | 42.7 |
| 2587 | H3 | I-II |  | WP_080997628.1 | 404 | 44.9 |
| 2588 | H4 | III |  | WP_049624660.1 | 386 | 41.4 |
| 2589 | H5 | III |  | WP_033016504.1 | 402 | 44.3 |
| 2590 | H6 | IV |  | WP_049625044.1 | 286 | 32.2 |
| 2591 | H7 | IV |  | WP_033010640.1 | 299 | 33.2 |
| 2592 | H8 | V |  | WP_033013843.1 | 360 | 40 |
| 2593 | H9 | V |  | WP_053532750.1 | 499 | 56.9 |
| 2594 | H10 | V |  | WP_033014262.1 | 370 | 40.6 |

**Supplementary Figure 1. Segment of the multiple sequence alignment of the thermophilic TA proteins,** highlighting (in purple boxes) the key conserved amino acid residues Asp259 and Lys288 in the active site (reference position number for CV2025).

**
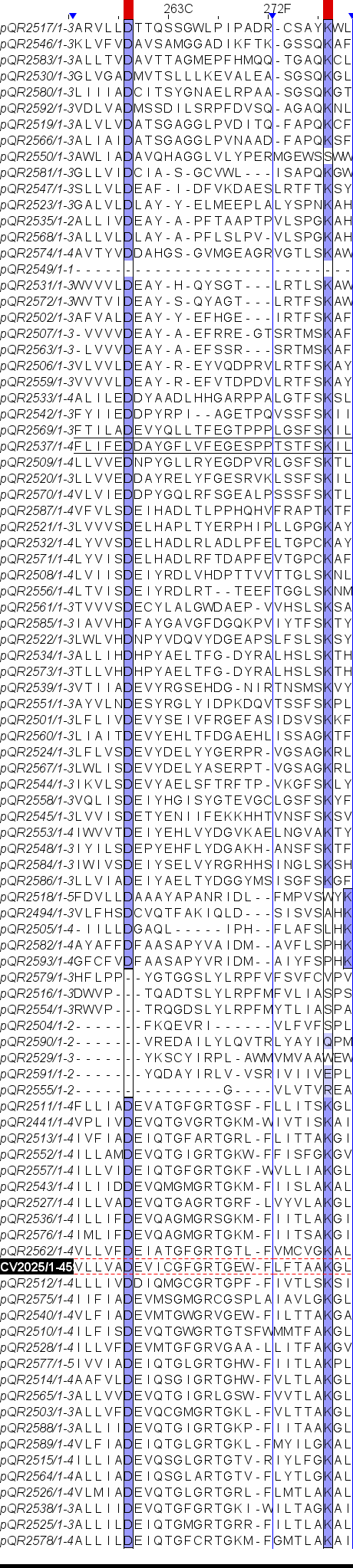
**

**Supplementary Figure 2. Segment of the multiple sequence alignment of thermophilic TAs class I-II,** highlighting (in coloured boxes) the conserved amino acid residue (reference position number for pQR2502).

**
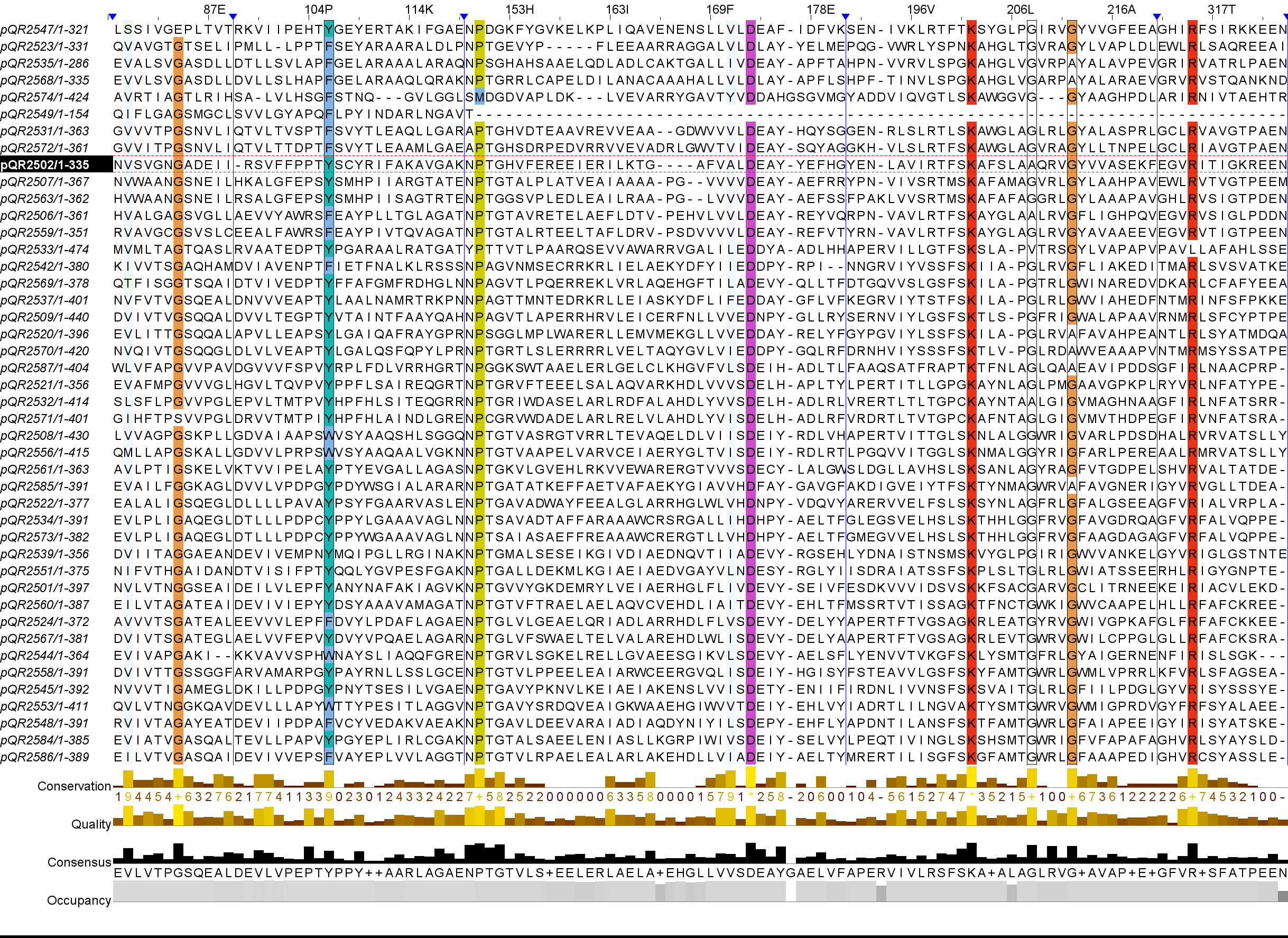
**

**Supplementary Figure 3. Segment of the multiple sequence alignment of thermophilic TAs class III,** highlighting (in coloured boxes) the conserved amino acid residues (reference position number for CV2025).

**
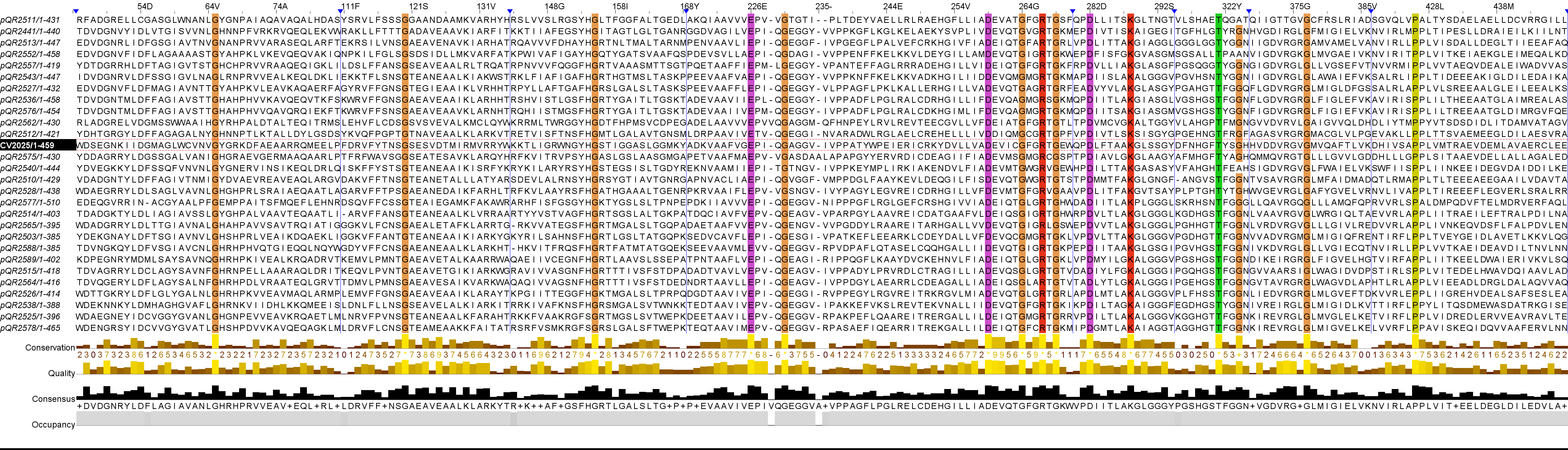
**


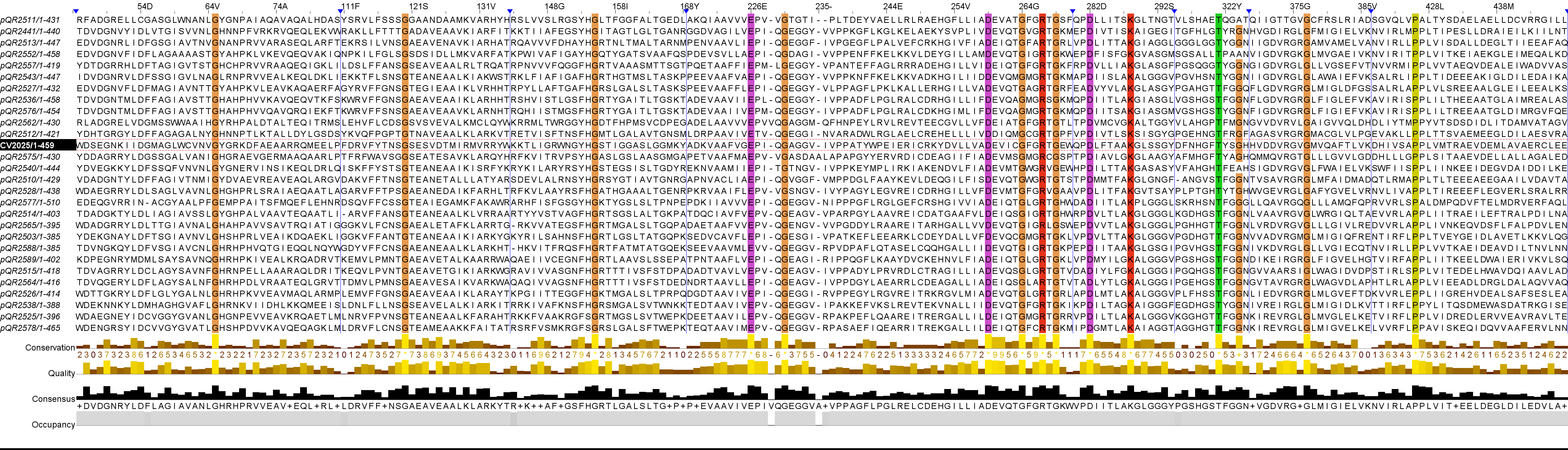

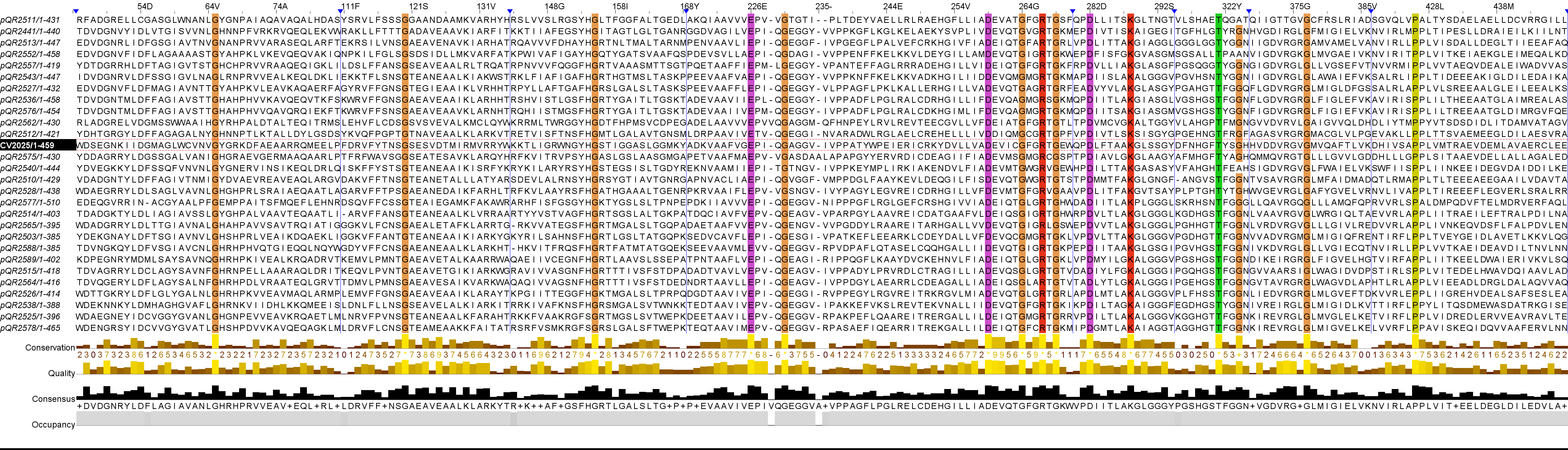


**Supplementary Figure 4. Protein sequence alignment of thermophilic TAs class IV,** highlighting (in coloured boxes) the conserved amino acid residues (based on position number for pQR2590). Cys/Thr243 equivalent to Lys288 (CV2025).

**
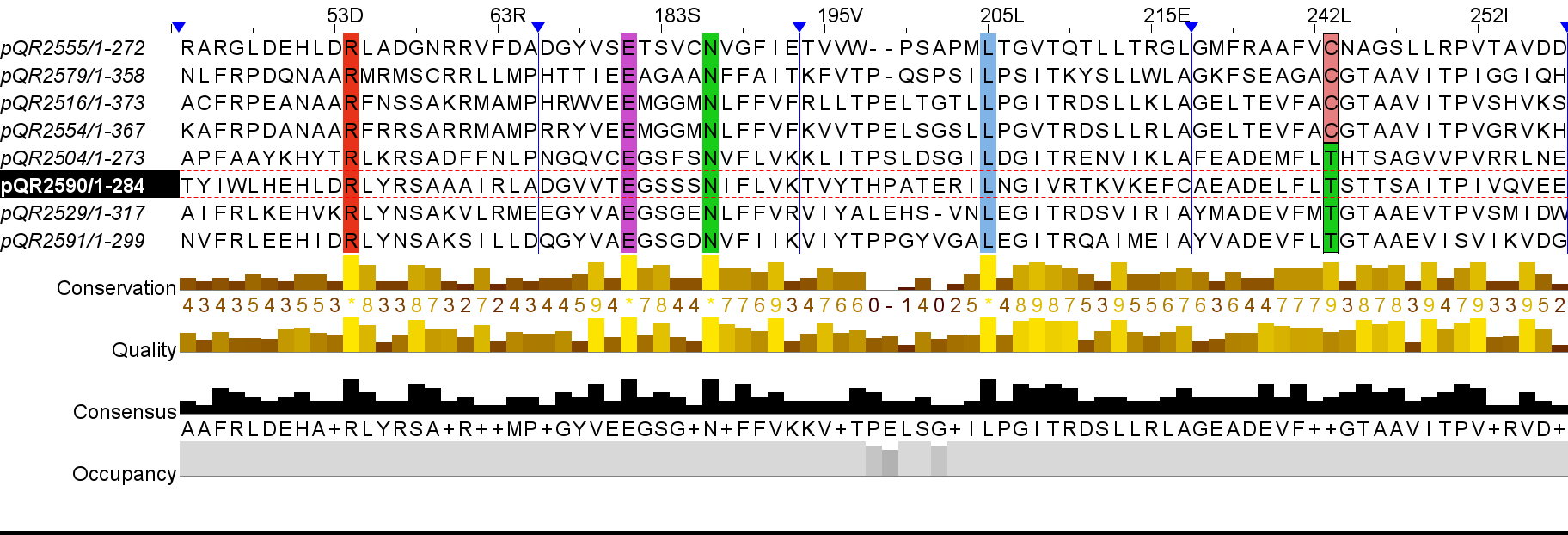
**

**Supplementary Figure 5. Segment of the multiple sequence alignment of thermophilic TAs class V.** Coloured boxed are the conserved amino acid residues (reference position for pQR2519).

**
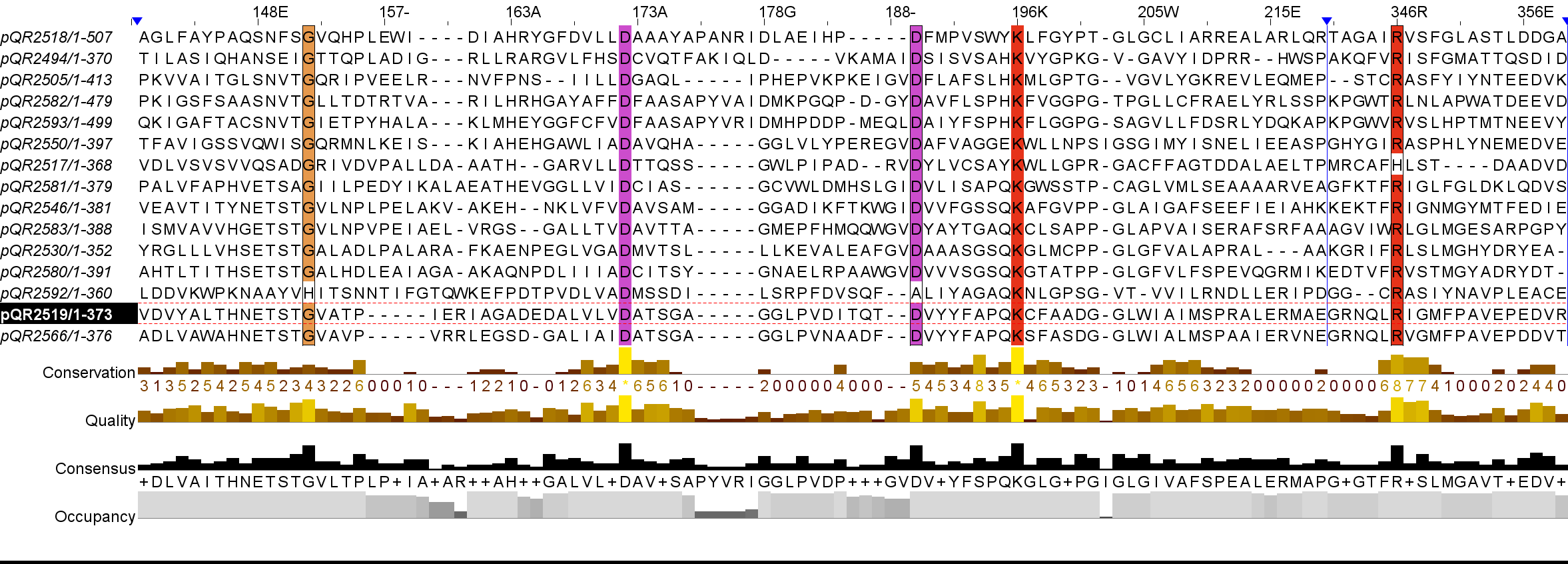
**

**Supplementary Figure 6. Expression of thermophilic TAs using a chemical chaperon media.** Media composition: Terrific broth, D-sorbitol 0.5 M, PLP 0.2 mM and phosphate buffer 0.1 M pH 7. Cultivation conditions: 1 mL of media per well at 1200 rpm at 37℃ until OD_600nm_ ~1.5; then induced with IPTG 0.5 mM and temperature reduced to 20℃ for additional 15 h. Label per lane represents the position in the plate panel.


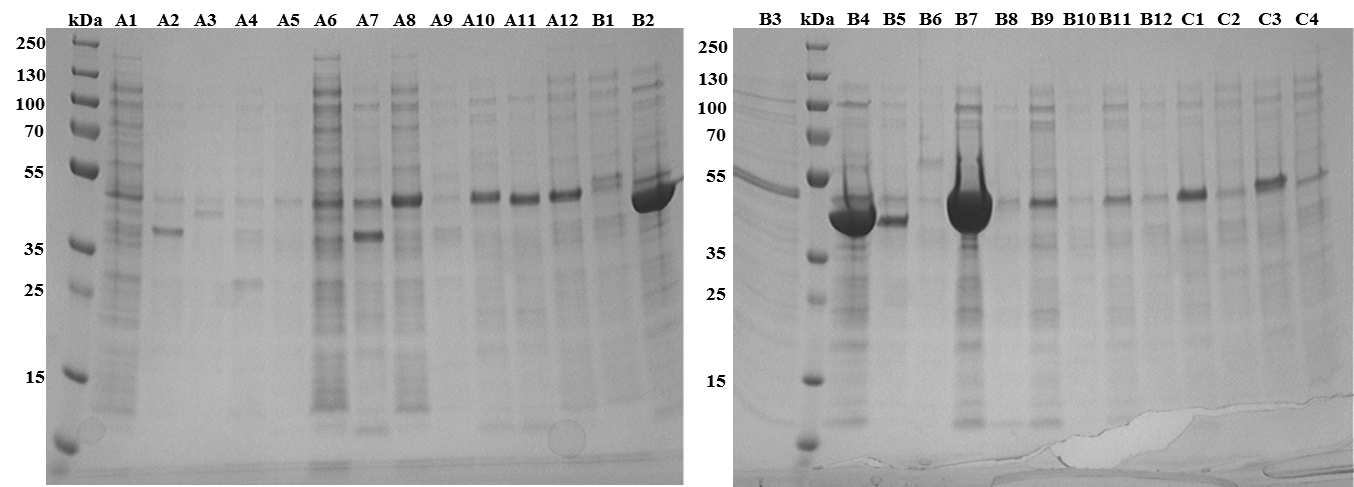


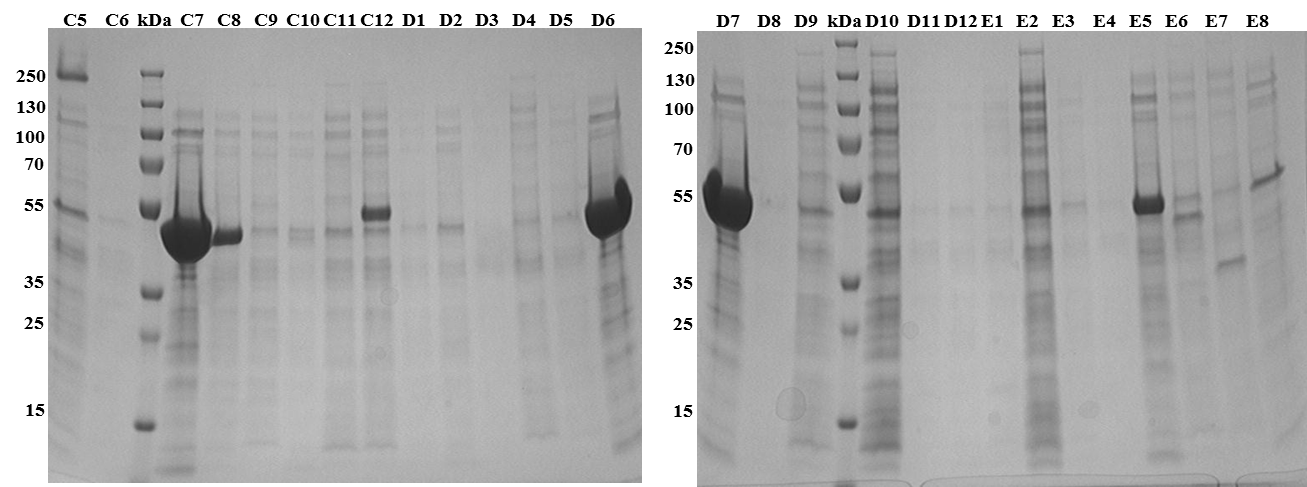


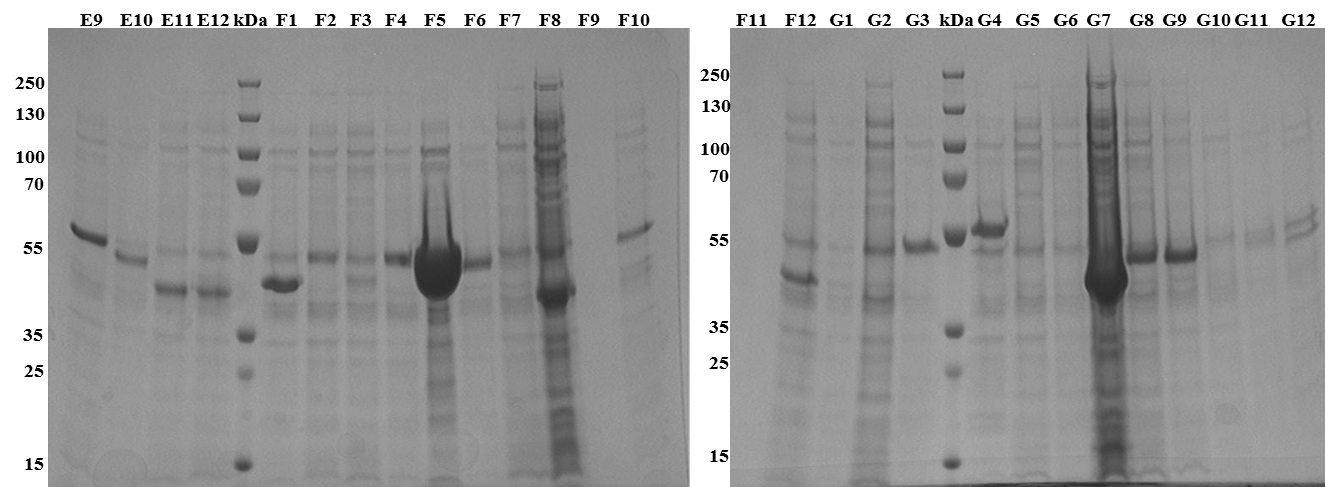


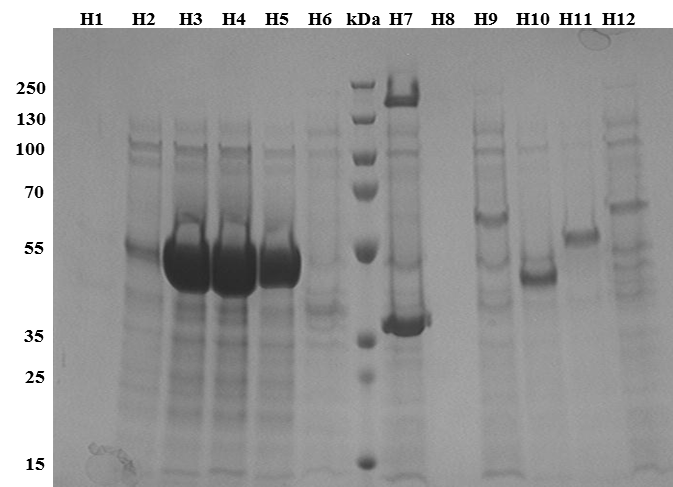


**Supplementary Figure 7. Chromatogram showing Hydroxypyruvate (HPA) and L-erythrulose (Ery).** HPLC analysis as explained in Section 2.7**.** Standards at 5 mM of each chemical.


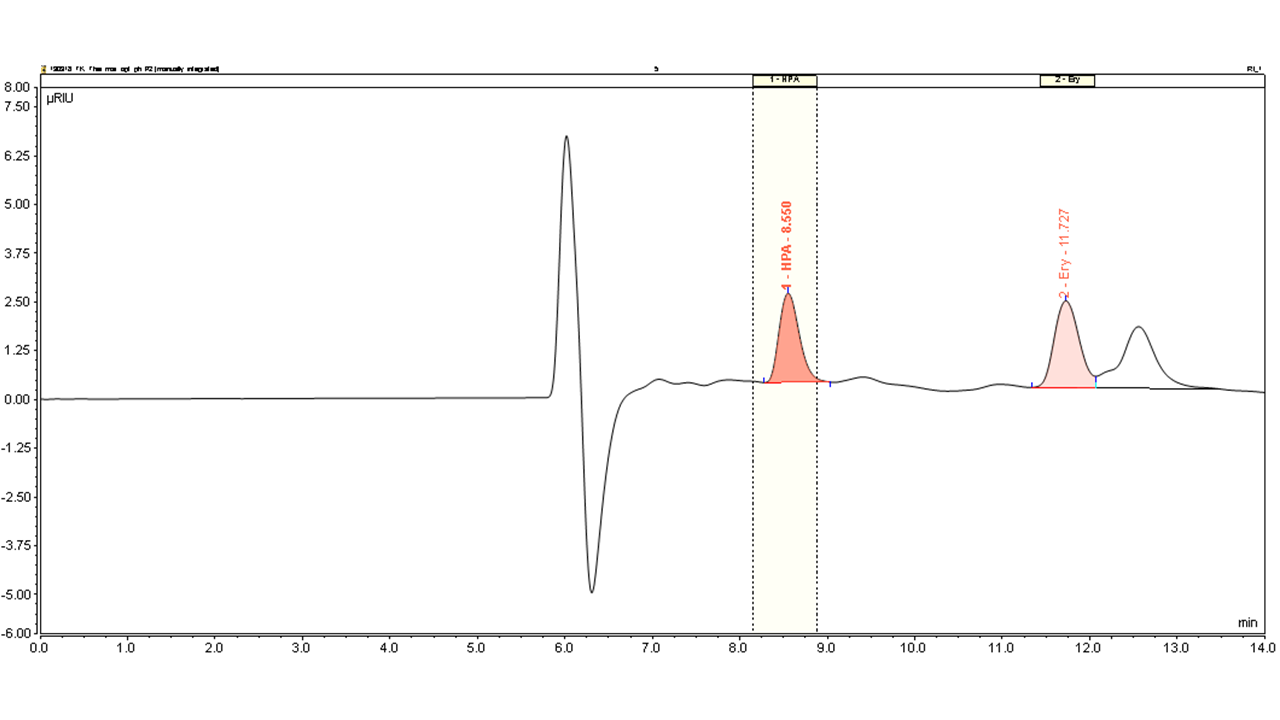

Supplement: Supplementary file 1 — Table S1. Thermophilic transaminase panel and pQR numbers (the Ward group plasmid identifier). Expression host system either E. coli BL21 (DE3) or (a) E. coli Rosetta 2 (DE3). Fig. S1. Segment of the multiple sequence alignment of the thermophilic TA proteins, highlighting (in purple boxes) the key conserved amino acid residues Asp259 and Lys288 in the active site (reference position number for CV2025). Fig. S2. Segment of the multiple sequence alignment of thermophilic TAs class I‐II, highlighting (in coloured boxes) the conserved amino acid residue (reference position number for pQR2502). Fig. S3. Segment of the multiple sequence alignment of thermophilic TAs class III, highlighting (in coloured boxes) the conserved amino acid residues (reference position number for CV2025). Fig. S4. Protein sequence alignment of thermophilic TAs class IV, highlighting (in coloured boxes) the conserved amino acid residues (based on position number for pQR2590). Cys/Thr243 equivalent to Lys288 (CV2025). Fig. S5. Segment of the multiple sequence alignment of thermophilic TAs class V. Coloured boxed are the conserved amino acid residues (reference position for pQR2519). Fig. S6. Expression of thermophilic TAs using a chemical chaperon media. Media composition: Terrific broth, D‐sorbitol 0.5 M, PLP 0.2 mM and phosphate buffer 0.1 M pH 7. Cultivation conditions: 1 mL of media per well at 1200 rpm at 37°C until OD600nm ~ 1.5; then induced with IPTG 0.5 mM and temperature reduced to 20°C for additional 15 h. Label per lane represents the position in the plate panel. Fig. S7. Chromatogram showing Hydroxypyruvate (HPA) and L‐erythrulose (Ery). HPLC analysis as explained in Section 2.7. Standards at 5 mM of each chemical. [file MBT2-15-305-s001.docx]
